# Gestational disruptions in metabolic rhythmicity of the liver, muscle, and placenta affect fetal size

**DOI:** 10.1096/fj.201601032R

**Published:** 2017-01-12

**Authors:** Georgia Papacleovoulou, Vanya Nikolova, Olayiwola Oduwole, Jenny Chambers, Marta Vazquez-Lopez, Eugene Jansen, Kypros Nicolaides, Malcolm Parker, Catherine Williamson

**Affiliations:** * Division of Women’s Health, Guy’s Campus, King’s College London, London, United Kingdom;; † Institute of Reproductive and Developmental Biology, Surgery and Cancer, Hammersmith Hospital, Imperial College London, London, United Kingdom;; ‡ Women’s Health Research Centre, Surgery and Cancer, Faculty of Medicine, Hammersmith Hospital, Imperial College London, London, United Kingdom;; § Centre for Health Protection, National Institute for Public Health and the Environment, Bilthoven, The Netherlands; and; ¶ Harris Birthright Centre for Fetal Medicine, King’s College London, London, United Kingdom

**Keywords:** circadian clock, metabolism, pregnancy, macrosomia, triglycerides

## Abstract

Maternal metabolic adaptations are essential for successful pregnancy outcomes. We investigated how metabolic gestational processes are coordinated, whether there is a functional link with internal clocks, and whether disruptions are related to metabolic abnormalities in pregnancy, by studying day/night metabolic pathways in murine models and samples from pregnant women with normally grown and large-for-gestational age infants. In early mouse pregnancy, expression of hepatic lipogenic genes was up-regulated and uncoupled from the hepatic clock. In late mouse pregnancy, rhythmicity of energy metabolism-related genes in the muscle followed the patterns of internal clock genes in this tissue, and coincided with enhanced lipid transporter expression in the fetoplacental unit. Diurnal triglyceride patterns were disrupted in human placentas from pregnancies with large-for-gestational age infants and this overlapped with an increase in BMAL1 expression. Metabolic adaptations in early pregnancy are uncoupled from the circadian clock, whereas in late pregnancy, energy availability is mediated by coordinated muscle-placenta metabolic adjustments linked to internal clocks. Placental triglyceride oscillations in the third trimester of human pregnancy are lost in large-for-gestational age infants and may be regulated by BMAL1. In summary, disruptions in metabolic and circadian rhythmicity are associated with increased fetal size, with implications for the pathogenesis of macrosomia.—Papacleovoulou, G., Nikolova, V., Oduwole, O., Chambers, J., Vazquez-Lopez, M., Jansen, E., Nicolaides, K., Parker, M., Williamson, C. Gestational disruptions in metabolic rhythmicity of the liver, muscle, and placenta affect fetal size.

In normal pregnancy, endocrine signals cause the maternal metabolic adaptations necessary to support the growing fetus, including enhanced storage of nutrients in the first 2 trimesters of human pregnancy (anabolic phase), and subsequent acceleration of transplacental nutrient transport (catabolic phase) to secure fetal growth and development (1, 2). We and others have shown gestational changes in hepatic lipid metabolism in humans and in rodents (2–6). Imbalance in nutrient availability and impaired transplacental transport pathways have been reported in intrauterine growth restriction and diabetic pregnancies (7, 8).

In mammals, there is a master pacemaker located in the suprachiasmatic nucleus (SCN) that synchronizes behavioral and physiologic rhythms in response to environmental cues [defined as zeitgeber time (ZT)]: activity/rest and feeding/nonfeeding cycles. Lipid homeostasis in peripheral tissues is tightly coupled to autonomous circadian systems that coordinate metabolic processes (9). Studies have demonstrated impaired metabolic homeostasis when circadian components in the SCN or periphery are blunted. *Clock^−/−^* mice develop obesity and metabolic syndrome, whereas disruption of *Bmal1* in white adipose tissue (WAT) impairs *de novo* lipogenesis in adipocytes (10, 11). This finding is consistent with the double *Clock/Bmal1*-knockout mouse model that shifts lipid accumulation to muscle and liver (12). In mice, Rev-erb-a and Rev-erb-b act as transcriptional corepressors that tightly control lipogenesis through regulation of the biosynthesis of fatty acid/triglyceride (FA/TG) and cholesterol. Their deficiency leads to hepatosteatosis (13–15), whereas administration of Rev-erb agonists improves dyslipidemia by increasing expression of genes involved in energy expenditure in the muscle (16).

Human epidemiologic studies have demonstrated a positive correlation between eating and sleeping patterns and shift work and features of metabolic syndrome (17–19). Moreover, shift workers have increased rates of adverse pregnancy outcomes (20, 21).

In the present study, we hypothesized that metabolic adaptations during the anabolic and catabolic phases of pregnancy are finely synchronized and are coordinated by internal clock genes. To address this hypothesis, we assessed the light–dark cycle (LDC) metabolic fluctuations in early and late pregnancy in mice and whether the alterations observed are tightly regulated by the peripheral clock machinery. Then, we investigated potential interrelations to the metabolic profile of the fetoplacental unit. To translate our findings to human disease, we also studied diurnal lipid fluctuations in human pregnancy, and investigated whether there are metabolic disruptions in placentas with large-for-gestational-age (LGA) infants.

## MATERIALS AND METHODS

### Animal studies

Age-matched (6- to 8-wk-old) female and male C57BL/6 inbred mice were purchased from Envigo (Derby, United) and maintained in a 24 h LDC (12/12 h) with free access to a normal chow diet (RM3; Special Diet Services, Essex, United Kingdom) and water. As described elsewhere (22), animals were allowed to acclimatize for a period of 2 wk and thereafter were mated on a ratio of 1 female with 1 male per cage. Daily inspection was made for copulation plugs, and when observed, the females were separated from the males. Pregnant animals were culled on d 7 (early; preplacentation) or d 14 (late; postplacentation) of pregnancy at 4-h intervals over a 12-h light–dark cycle [*n* = 5–7 animals per gestational day per time point; light cycle ZT24, ZT4 and ZT8 and dark cycle; *i.e.,* ZT12, ZT16, and ZT20]. Supplemental Fig. S1*A* illustrates how d 7 and 14 of murine pregnancy reflected 2 separate phases of gestation; preplacentation (early organogenesis) and postplacentation (fetal growth and development) that correspond to early (trimesters 1 and 2) and late (third trimester) human pregnancy (23). Moreover, d 14 was when triglyceride levels started to increase in pregnant mice, delineating a metabolic switch (Supplemental Fig. S1*B*). Animals were culled under red light during the dark phase (24). To avoid potential disparities in metabolic profile related to different phases of the estrous cycle (25, 26), mice that were euthanized 1 d after identification of a copulation plug served as nonpregnant controls (nonestablished gestation). Maternal gonadal WAT, skeletal muscle, placenta, serum, and maternal and fetal liver were collected for analysis. All experimental procedures were approved by the ethics committee for animal welfare at Imperial College London, and all animal studies were performed in accordance with the UK Animals (Scientific Procedures) Act of 1986 and the guidelines from the biologic sciences unit at Imperial College London.

### Human studies

We measured serum cholesterol and triglycerides in pregnant (*n =* 7) nonobese and nondiabetic women, before and after a standardized meal (containing 100 g carbohydrates and 50 g fat; total energy, 950–1083 kcal) and we compared them to nonpregnant parous women (*n =* 4). Pregnant women carried infants of a mean gestational age of 33 ± 1.13 wk). A blood sample was collected at 8 am after an overnight fast, and breakfast was provided at 9:00 am. Blood samples were collected immediately after breakfast and at 11.45 am. Lunch was provided at 12 pm, and additional blood samples were collected at 1, 2, and 3 pm. All women gave informed consent, and the study was approved by the local ethics committee of Hammersmith Hospital (11/L0/0396).

### Human placenta

Samples of human term placenta were obtained from the Baby Bio Bank, University College London (Project 524578.100.156822) from women with no metabolic disease of pregnancy who had elective cesarean section and gave birth to normal-size [50–75th percentile; control; *n =* 38; mean gestational age 38 ± 0.2 wk; mean body mass index (BMI) 24 ± 0.7] or LGA (>95th percentile; *n =* 37; mean gestational age 38 ± 0.3 wk; mean BMI 28 ± 0.9) infants. Samples were obtained at the following time points (*n =* 4–8 per group): 9–11 am, 11 am–1 pm, 1–3 pm, 3–5 pm, and 9 pm–12 am. All patients gave informed consent, and the ethics of the study protocol were approved (08/H0707/21).

### Biochemical measurements

Serum and tissue biochemical parameters [cholesterol, triglycerides, and free fatty acids (FFAs)] were measured, with an LX20 autoanalyzer (Beckman Coulter, Brea, CA, USA), as described in Papacleovoulou *et al*. (27). Serum triglyceride and cholesterol levels were measured in samples from the standardized metabolic feeding study at the Hammersmith Hospital chemical pathology laboratory.

### Real-time quantitative PCR

Total RNA from mouse liver, muscle, gonadal WAT, placenta, fetal liver, and human placenta was processed (27). Primer sequences (Sigma-Aldrich, Poole, United Kingdom) are provided in Supplemental Table S1.

### Statistical analysis

All data sets were combined and presented as means ± sem. Statistical analysis for multiple comparisons was performed by repeated-measures ANOVA and Newman-Keuls *post hoc* testing with Prism 7.00 software (GraphPad Software, La Jolla, CA, USA). For single comparisons in human samples (Supplemental Table S2) nonparametric, the 2-tailed Mann-Whitney *U* test was used. The significance cutoff was set at *P* ≤ 0.05.

## RESULTS

### Fluctuations of serum lipids during the LDC in pregnancy

**Figure 1.**
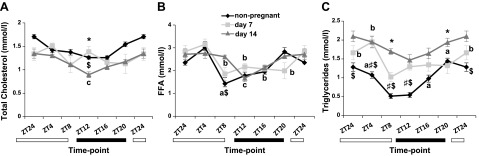
Serum lipid oscillations during the LDC in mouse pregnancy. Serum from d 7 and d 14 pregnant and nonpregnant female mice was assessed for total cholesterol (*A*), FFAs (*B*), and triglycerides (*C*). Data are means ± sem (*n* ≥ 5 per group per time point). *^a–c^P* < 0.05; nonpregnant (*a*), d 7 (*b*), d 14 (*c*) for fluctuations during LDC within the same stage of pregnancy. ^*^P < 0.05 for d 7 *vs*. 14, ^#^*P* < 0.05 for nonpregnant *vs*. d 7, ^$^*P* < 0.05 for nonpregnant *vs*. d 14 for comparisons at the same ZT point in different stages of pregnancy.

As it has been shown that serum lipids fluctuate during the LDC in mice (28), we investigated LDC oscillations on d 7 and 14 of pregnancy compared with those in nonpregnant controls. Total cholesterol levels did not vary significantly between nonpregnant and pregnant animals, although on d 14 of pregnancy, there was a drop at the beginning of the dark cycle (ZT12; **Fig. 1*A***). Serum FFA levels did not differ between d 7 and 14 pregnant animals, and they fluctuated over the LDC (Fig. 1*B*). Triglyceride concentrations were increased throughout the day in late pregnancy and did not fluctuate within the LDC, as seen on d 7 (Fig. 1*C*).

### Regulation of lipid metabolism in mouse pregnancy

**Figure 2.**
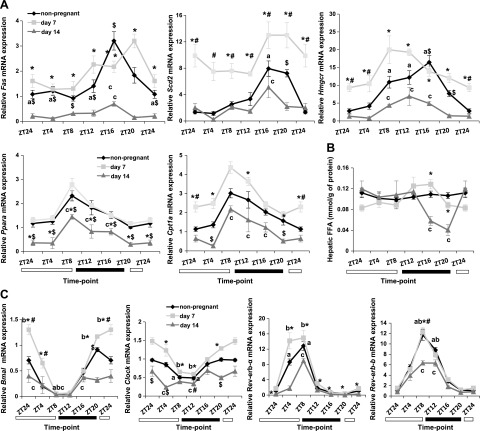
Metabolic and circadian gene expression and endogenous FFA levels of pregnancy during the LDC in the liver. *A*) Hepatic transcriptional profile of early pregnant (d 7), late pregnant (d 14), and nonpregnant control mice for *Fas*, *Scd2*, *Hmgcr*, *Ppara*, and *Cpt1a* genes. *B*) Endogenous FFA levels in the liver. *C*) Gene expression patterns of clock genes. Data are means ± sem (*n* ≥ 5 per group per time point). *^a–c^P* < 0.05; nonpregnant (*a*), d 7 (*b*), d 14 (*c*) for fluctuations during LDC within the same stage of pregnancy. ^*^P < 0.05 for d 7 *vs*. 14, ^#^*P* < 0.05 for nonpregnant *vs*. d 7, ^$^*P* < 0.05 for nonpregnant *vs*. d 14 for comparisons at the same ZT point in different stages of pregnancy.

It is well established that metabolic transcriptional machinery oscillates during the LDC in murine liver and muscle (22). We tested whether metabolic pathways are differentially regulated during mouse pregnancy. Hepatic lipogenic genes (*Fas*, *Scd2*, and *Hmgcr*; **Fig. 2*A***) were expressed at significantly higher levels on d 7 of pregnancy when compared to d 14. On d 7, *de novo* lipogenic genes were expressed at higher levels throughout the LDC. In contrast, despite their reduced expression levels, the LDC oscillations were maintained on d 14 of pregnancy. *Fas* and *Scd2* mRNA peaked at ZT16, whereas *Hmgcr* mRNA peaked just before the dark cycle and stayed up-regulated until ZT16. Consistent with the negative feedback of FA biosynthesis (29), the increased mRNA of the *Fas* and *Scd2* genes at ZT16 on d 14 of pregnancy was accompanied by significantly decreased FFA levels in the liver at ZT16, with no fluctuations in d 7 pregnant animals (Fig. 2*B*). Similar to the hepatic lipogenesis profile, fatty acid oxidation genes (*Ppara* and *Cpt1a*; Fig. 2*A*) were also expressed at higher levels on d 7 compared to d 14 of pregnancy. Nonetheless, on d 14 of pregnancy, *Ppara* and *Cpt1a* mRNA levels oscillated during the LDC, with a peak at ZT8.

It has been demonstrated that lipogenesis is coupled to oscillations entrained in the cell autonomous clock in the liver (13). To see whether this relation is maintained in pregnancy, we evaluated the gestational transcriptional profile of hepatic clock genes. Consistent with metabolic genes (**Fig. 2*A***), mRNA expression levels of the hepatic clock genes *Bmal1*, *Clock*, *Rev-erb-a*, and *Rev-erb-b* were significantly higher at least at 1 time point on d 7 compared with d 14 levels. Nevertheless, no differences in the patterns of LDC rhythmicity were observed in *Bmal1* and *Clock* genes in the different stages of pregnancy (Fig. 2*C*). Similar to nonpregnant animals, hepatic lipogenic genes (Fig. 2*A*, top) peaked when *Rev-erb-a* and *Rev-erb-b* mRNA expression declined in d 14 pregnant animals, whereas on d 7 of pregnancy, the lipogenesis gene mRNA profile was not coupled to the daily rhythms of the hepatic clock gene machinery (Fig. 2*C*).

Another tissue that regulates lipid homeostasis is WAT. In gonadal WAT, lipogenic genes did not differ in mRNA levels and did not fluctuate during the light–dark cycle (Supplemental Fig. S2*A*). No profound differences were observed in clock gene rhythmic patterns in WAT (Supplemental Fig. S2*B*).

### Regulation of energy homeostasis in mouse pregnancy

**Figure 3.**
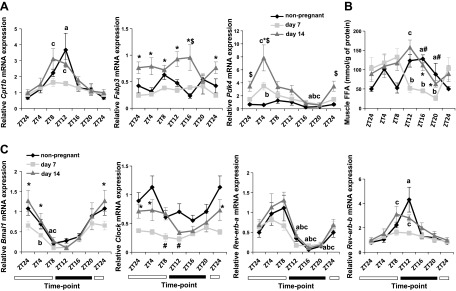
Metabolic and circadian gene expression and endogenous FFA levels of pregnancy during the LDC in the muscle. *A*) Transcriptional profile of the energy homeostasis genes *Cpt1b*, *Fabp3*, and *Pdk4* in muscle of early pregnant (d 7), late pregnant (d 14), and nonpregnant control mice. Endogenous FFA levels (*B*). Gene expression of clock genes (*C*). Data are means ± sem (*n* ≥ 5 per group per time point). *^a–c^P* < 0.05; nonpregnant (*a*), d 7 (*b*), d 14 (*c*) for fluctuations during LDC within the same stage of gregnancy. ^*^P < 0.05 for d 7 *vs*. 14, ^#^*P* < 0.05 for nonpregnant *vs*. d 7, ^$^*P* < 0.05 for nonpregnant *vs*. d 14 for comparisons at the same ZT point in different stages of pregnancy.

To assess energy availability for maintenance and development of the fetus, we investigated energy uptake– and expenditure-related genes in the muscle during mouse pregnancy. mRNA of the fatty acid oxidation rate-limiting gene, *Cpt1b* peaked at ZT8 and -12 in d 14 pregnant mice. mRNA expression of the fatty acid binding gene *Fabp3* was significantly up-regulated on d 14 compared to d 7; however, no altered rhythmicity was noted (**Fig. 3*A***). Moreover, the glucose oxidation gene, *Pdk4*, oscillated at ZT4 on both d 7 and 14 of pregnancy, and gene expression increased at ZT4 on gestational d 14 compared to d 7. A peak in muscle FFA concentrations at ZT12 was observed on d 14 of pregnancy (Fig. 3*B*). Overall, we observed an increased metabolic activity of the muscle on d 14 of pregnancy compared to d 7. We also tested whether energy uptake and breakdown in pregnancy have a circadian component as they do outside pregnancy (22, 30, 31). Like energy homeostasis genes, the mRNA expression of the *Bmal1* and *Clock* genes was lower on d 7 compared to d 14, at least in the light phase of the LDC. In addition, on d 7 of pregnancy, *Bmal1* mRNA levels dropped at ZT4 as opposed to ZT8 on d 14 of pregnancy (Fig. 3*C*). Furthermore, oscillations in *Clock* mRNA were down-regulated on gestational d 7, although they did not reach significance on either gestational d 14 or in nonpregnant animals. Rhythmic patterns of *Rev-erb-a* were maintained in both pregnant and nonpregnant animals in the muscle. Similar to *Cpt1b* (Fig. 3*A*), *Rev-erb-b* oscillation was shifted from ZT12 to ZT8 on d 14 of pregnancy compared to nonpregnant controls, whereas on d 7, *Rev-erb* oscillation was blunted (Fig. 3*C*). These data demonstrate that muscle coordinates energy uptake and availability later in pregnancy in a process mediated by *Rev-erb-b*.

### Regulation of transplacental nutrient transport in mouse pregnancy

**Figure 4.**
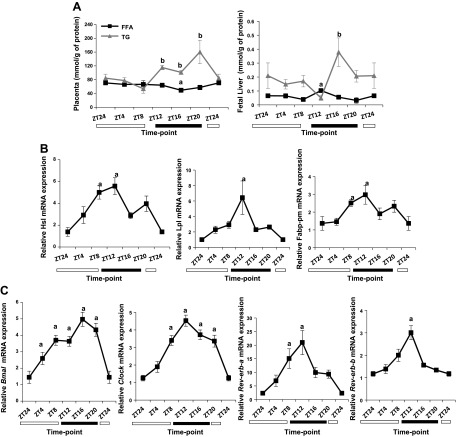
Transplacental nutrient transport during the LDC. *A*) FFA and TG concentrations in placenta and fetal liver on d 14 of pregnancy. *B*) Gene expression profile of lipases and fatty acid transport on d 14 of pregnancy. *C*) Gene expression of clock genes during LDC in placenta. Data are means ± sem (*n* ≥ 5 per group per time point). ^*a*, *b*^*P* < 0.05; fluctuations of FFA (*a*) and TG (*b*) during LDC.

We hypothesized that the increased *Cpt1b* expression at ZT8 (Fig. 3*A*), followed by raised muscle and reduced circulating FFA levels at ZT12 (Figs. 3*B*, 1, respectively), is synchronized with transplacental nutrient transport. To address this, we measured lipid concentrations in placenta and fetal liver, and we evaluated expression of genes that are involved in FA/TG transport. Whereas FFA levels did not fluctuate, either in placenta or fetal liver on d 14 of pregnancy, triglyceride levels were elevated during the dark phase (ZT12–20) in placenta and peaked at ZT16 in the fetal liver (**Fig. 4*A***). Accordingly, placental lipases (Hsl and Lpl) as well as fatty acid–binding (Fabp-pm) mRNA peaked at the end of the light phase (ZT8) or at the beginning of the dark phase (Lpl; ZT12) (Fig. 4*B*). Placenta clock genes were also present, with cyclical changes in mRNA levels during the LDC (Fig. 4*C*). *Rev-erb-a* and *Rev-erb-b* peaked at ZT8 and -12, respectively.

### Regulation of lipid homeostasis in human pregnancy

**Figure 5.**
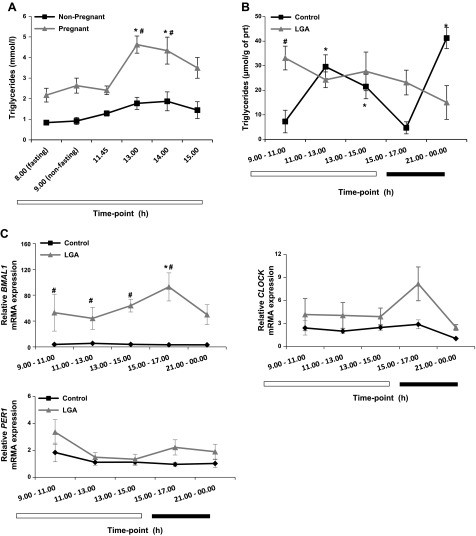
Triglyceride levels and clock gene expression patterns during the day in human pregnancy. *A*) Triglyceride levels in the serum of pregnant and nonpregnant women after an overnight fast followed by a standardized high-calorie meal. Mean gestational age, 33.1 ± 1.13 wk. Data are means ± sem. *P < 0.05 for fluctuations during the day; ^#^*P* < 0.05 for pregnant *vs.* nonpregnant women. *B*) Diurnal fluctuations of triglyceride levels in normal pregnancy are not maintained in LGA pregnancy. *C*) Clock gene mRNA expression profile in human placenta. *BMAL1* (left) has increased mRNA levels in LGA pregnancy compared to controls. No changes were observed in *CLOCK* (right) or *PER1* (bottom) mRNA. Data are means ± sem (*n* = 4–8 per group per time point). *P < 0.05 for fluctuations during the day; ^#^*P* < 0.05 for differences in gene expression levels.

To investigate whether there are any lipid fluctuations in human pregnancy, we measured serum cholesterol and triglycerides in pregnant and nonpregnant parous women before and after a standard high-calorie meal in the morning and afternoon. Although there was no change in lipids of the nonpregnant women after the meals, the pregnant women showed a significant increase in serum triglyceride levels after lunch (**Fig. 5*A***). To further study diurnal fluctuations in human pregnancy and whether these are relevant to LGA infants (>95th percentile), we used placentas from elective caesarean sections collected at different times of the day (*n* ≥ 5 per group per time point). The BMI of women who gave birth to LGA infants was significantly higher (LGA, 28 ± 0.9 *vs.* control 24 ± 0.7), at least when they first visited the clinic, consistent with previous studies (32–34). No differences were observed in gestational age at delivery (Supplemental Table S2). Triglyceride levels fluctuated in placentas of normal pregnancies with a peak at the 11 am to 1 pm and late-night time points (Fig. 5*B*), whereas no significant fluctuations in triglyceride levels were observed in LGA placentas, in which triglyceride concentrations were significantly increased in the morning and remained elevated. To assess the dynamics of metabolic processes in placenta between early and late human pregnancy, we also collected chorionic villus sampling (CVS) specimens (collected between 9 and 14 gestational week) at different times of the day (*n* ≥ 3 per time point). We compared expression levels of lipid transport and clock genes in early pregnancy (CVS) and third-trimester placentas (elective caesarean sections) and whether this is affected in LGA. No fluctuations were observed in clock or lipid transport genes in early pregnancy (CVS) (Supplemental Fig. S3*A*–*C*). *BMAL1*, *CLOCK*, and *PER1* were detected in term placentas, but those genes did not oscillate in control pregnancy (Fig. 5*C*). However, *BMAL1* mRNA was expressed at elevated levels in LGA, and a trend toward an increase of *CLOCK* mRNA was shown at the 3 to 5 pm time point. The nutrient transport-related genes, *CD36* and *LAL* did not change in normal pregnancy, but there was a trend for daily fluctuations of *CD36* in LGA placentas (Supplemental Fig. S4).

## DISCUSSION

This study reveals reciprocal changes in lipid homeostasis pathways between peripheral tissues at different stages of mouse and human pregnancy. Our data indicate that maternal adaptations in mouse pregnancy were coordinated by synchronization of metabolic processes in liver, skeletal muscle, and placenta, and these were linked to altered circadian signals. Early pregnancy was associated with a sustained increase in hepatic lipogenesis uncoupled from the circadian clock, whereas there was a down-regulation of hepatic lipogenic genes in the last third of mouse pregnancy. Furthermore, hepatic LDC rhythmicity was preserved on gestational d 14 and coincided with the negative feedback oscillations of *Rev-erb-a* and *Rev-erb-b*. When hepatic lipogenesis was down-regulated on d 14 of pregnancy, there was an increase in glucose and fatty acid oxidation in the skeletal muscle. Notably, in the muscle, FFA levels dropped during the dark phase of the cycle when triglyceride levels in the placenta and fetal liver increased. This increase coincided with a peak expression of lipases and fatty acid transporters in placenta, implying a role for muscle in nutrient availability for transplacental transfer to the fetus. These changes reflect oscillations of the peripheral clock genes in the placenta. To address these concepts in human pregnancy, we used clinical samples obtained from CVS procedures (mean gestational age, 11.5 wk) to delineate early pregnancy events, and term placentas from elective cesarean sections (mean gestational age, 38 wk) to investigate late pregnancy events. Moreover, we studied serum lipid levels in pregnant women after standard meals and compared them with those of nonpregnant women. We found an acute postprandial increase in serum triglyceride levels in third-trimester pregnant women after a high-calorie lunch compared to levels in nonpregnant women. Furthermore, although placental triglyceride levels were subject to diurnal oscillations in the third trimester of an uncomplicated pregnancy, it was disrupted in LGA cases, where placental triglyceride concentrations were consistently elevated. No profound alterations in daily patterns of clock genes or lipid transport pathways were observed in CVS samples.

The liver governs whole-body energy metabolism, because it is the master regulator of energy production, storage, and release and provides the substrates that can be subsequently utilized by extrahepatic tissues such as WAT and skeletal muscle (35). It is well established that the liver undergoes metabolic adjustments to maintain pregnancy and promote growth of the fetus (3). The first phase of pregnancy is a metabolically active state, when the body has to accumulate and store substrates to fulfill fetal demands (2). Our data established that on d 7 of murine pregnancy, the expression levels of hepatic lipogenic genes, such as *Fas*, *Scd2*, and *Hmgcr*, fatty acid oxidation genes, such as, *Ppara* and *Cpt1a*, were increased compared to d 14, and this increase was not coupled with the cell-autonomous clock system of the liver. A similar uncoupling of the internal clock system has been demonstrated in the mammary gland during lactation, another period of high-energy demand in the female’s life (36). In addition, daily rhythms of core body temperature were demonstrated to be blunted in pregnancy (37). In contrast, albeit with reduced gene expression levels, hepatic circadian oscillations of metabolic and clock genes were maintained on gestational d 14, and this concurred with recent findings (38). On d 14, hepatic *de novo* lipogenesis followed the negative-feedback oscillations of *Rev-erb-a* and *Rev-erb-b*, consistent with studies of these corepressors outside pregnancy (13). These data indicate that the liver undergoes unique temporal adjustments in early and advanced gestation. A constant lipid synthesis and storage output on d 7 of pregnancy during the LDC in the liver was replaced by an oscillating “switch-on” and “switch-off” of lipid synthesis, storage, and oxidation on d 14. This process suggests a tight control and commitment of the liver to maintain nutrient availability in pregnancy.

Given the differential hepatic activities in lipid homeostasis between early and late pregnancy, we investigated how the stored energy is released and transferred to the fetus. WAT and muscle are responsible for energy uptake and release. The oscillation patterns in clock genes and lipid homeostasis genes of gonadal WAT were maintained in pregnancy. This is not consistent with data from a recent study that demonstrated that gonadal WAT rhythmicity of metabolic genes is associated with rhythms of the circadian clock and that pregnancy is decoupled from oscillations (37). This discrepancy may be explained by differences in gestational days studied and methods used to maintain and cull the animals. However, our data indicated that muscle has an important role in maternal adaptations of pregnancy, especially in the catabolic gestational phase when transplacental lipid and nutrient transport is enhanced. This is a novel concept in maternal adaptations of pregnancy. Muscle has a major role in energy homeostasis as it breaks down glycogen and proteins and releases lactate and alanine (35). Furthermore, fatty acid oxidation in the liver is essential for synthesis of ketone bodies, as well as release of other energy substrates to the bloodstream, all of which contribute to fetal growth (39). On d 14 of pregnancy, expression of genes involved in fatty acid oxidation pathways in the liver (*Cpt1a* and *Ppara*) and muscle (*Cpt1b*) peaked at ZT8, followed by oscillations of the lipid transport pathways in the placenta (ZT8 and ZT12). In parallel, the glucose oxidation gene *Pdk4* peaked at ZT4 in the muscle on gestational d 14, consistent with its role in facilitating fatty acid oxidation for energy release (40). This overlapped with accumulation of triglycerides during the dark phase in the placenta and fetal liver. Moreover, we showed that energy-balance–associated genes in the muscle were expressed at lower levels on d 7 compared with d 14, with minimal or no oscillation patterns. *Cpt1b* gene expression oscillated with a similar pattern to *Rev-erb-b* on gestational d 14, whereas it was blunted on d 7. These data imply a role of muscle in programming the energy availability for the fetoplacental unit. At the same time, although hepatic lipogenesis was partially blunted on d 14, hepatic fatty acid oxidation appeared to be active, indicating temporal reprogramming of the liver to provide energy resources, most likely ketone bodies. This finding is consistent with the known susceptibility of pregnant women to ketoacidosis in the third trimester (41).

Remarkably, and unlike mouse pregnancy, we did not detect any oscillation patterns in clock genes in term human placentas, which is not consistent with previous studies of placentas from vaginal deliveries (42). This discrepancy may be because we used placentas from elective cesarean sections. No oscillation patterns were observed in clock or metabolic genes of CVS specimens collected early in pregnancy. Similar to mouse pregnancy, this result agrees with the anabolic phase of early gestation that is characterized by increased lipid synthesis and storage, and less with transport of nutrients to the fetus.

In the present study, we revealed an acute postprandial increase in the serum triglyceride levels in pregnant women that was not observed in controls, and this was consistent with previous reports (43). This increase was noted after lunch but not after breakfast, and it may be a response to overnight fasting. It is very likely that FFAs are acutely increased after overnight fasting, as has been described (43). We also demonstrated a diurnal pattern in placental triglyceride levels that was disrupted in LGA pregnancies. Maternal hypertriglyceridemia has been demonstrated in LGA pregnancies, even in normoglycemic women (33). Our data imply that continuously increased concentrations of triglycerides in LGA placentas may contribute to excess breakdown of the latter into FFAs that, in turn, are transported to the fetal circulation, thereby enhancing fetal growth. Indeed, studies that were conducted to correlate maternal hypertriglyceridemia with macrosomia have shown raised fasting triglyceride levels in the first and third trimesters of LGA pregnancies (33, 34). This association was independent of prepregnancy BMI, which is also reported in mothers of LGA infants (33). In the present study, we did not see differences in gene expression levels or fluctuations of the fatty acid transporter, *CD36* or the cytosolic lysosomal acid lipase between normal and LGA placentas. However, diurnal patterns of gene expression levels do not necessarily reflect the extent of nutrient transport, because the latter is also regulated by facilitated diffusion, active transport against concentration gradients, and it is also highly dependent on placental size and fetoplacental blood flow (reviewed in ref. 44). The elevated maternal triglyceride concentrations after lunch in uncomplicated pregnancies in conjunction with persistent elevated placental triglyceride levels in LGA placentas is likely to be of clinical relevance, given that LGA infants of nondiabetic mothers are at increased risk of hypoglycemia, hypoxia, shoulder dystocia, and plexus injuries and have greater need for intensive care (32). Moreover, the raised triglyceride levels observed in LGA placentas were associated with up-regulated expression of the clock gene *BMAL1*. It is well established that disruption of *Bmal1* in WAT impairs *de novo* lipogenesis in adipocytes (10, 11), whereas, in the double *Clock/Bmal1*-knockout mouse model, lipid accumulation shifts to muscle and liver (12). Thus, it is plausible that the increase in placental *BMAL-1* promotes triglyceride accretion in placenta that can lead to LGA infants.

Murine and human pregnancy are characterized by increased lipid synthesis in the first two-thirds of gestation and gradual elevation of serum triglycerides as pregnancy progresses (39, 45, 46). However, discrepancies have been noted in maternal cholesterol levels (Supplemental Fig. S1*B*). Unlike human pregnancy, in mouse pregnancy, there is a gradual drop in maternal cholesterol levels of unfed mice from d 7 of pregnancy that is more profound closer to term. This effect is most likely explained by the fact that the mouse fetus can perform *de novo* cholesterol biosynthesis toward the end of pregnancy, whereas in human pregnancy, a significant proportion of fetal cholesterol originates from the mother (39, 47, 48). It should be noted that in the current study, food intake was not monitored, and patterns of lipid levels and gene expression during the light–dark cycle were assessed in fed mice. In contrast, in our human pregnancy data, women fasted for at least 8 h before undergoing cesarean section and in the case of serum lipid measurements, the participants had a controlled diet. Nonetheless, using the findings of our mouse model of pregnancy, we were able to establish which clinical samples to collect and the stage of human gestation that was most appropriate to study, to understand alterations in metabolic activity in normal and potential disruptions in pathologic pregnancy. Our human studies were limited because of the inability to obtain CVS specimens during the night, and we were unable to evaluate muscle metabolism in pregnant women.

The dynamic changes in the liver and muscle metabolic processes during pregnancy observed in the present study are also relevant to gestational carbohydrate metabolism, given that glucose is the principal energy substrate used by the fetus; and therefore, maternoplacental adaptations in glucose metabolism are essential to secure fetal glucose demands (49, 50). Diurnal fluctuations of glucose with nocturnal hypoglycemia has been demonstrated in the third trimester of human pregnancy (43, 51) and abnormalities in insulin responses have been noted in women at high risk of developing gestational diabetes mellitus (GDM) (52). In mouse pregnancy, the importance of glucose and insulin dynamics has also been established, especially toward the time of delivery and is fundamental, not only for successful pregnancy outcomes but also for the subsequent health of the offspring (53). Oscillations of genes associated with glucose homeostasis were shown to decrease in the liver of animals in late pregnancy, and this phenotype is related to a decrease in oscillations of hepatic clock genes, emphasizing the importance of glucose homeostasis adaptations to fulfill fetal demands (38). Although in the current study the pregnant women who gave birth to LGA infants were not diabetic, they had increased BMI as well as placenta hypertriglyceridemia. We cannot therefore exclude the possibility that this phenotype is associated with dysregulation of glucose homeostasis in LGA pregnancies, as seen in GDM and macrosomia (54).

**Figure 6.**
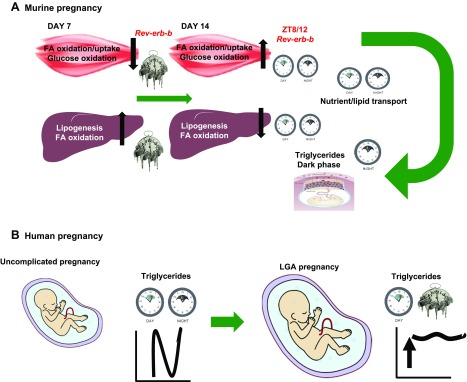
Daily rhythms in circadian and metabolic processes in pregnancy. *A*) The liver–muscle–placental gestational switch. Metabolic adaptations are tightly programmed in mouse pregnancy. Hepatic genes involved in metabolic processes show constantly higher expression levels on d 7 of pregnancy, followed by a drop in gene expression levels on d 14. Day 7 hepatic metabolism is uncoupled from the circadian clock (represented by the melted clock image), whereas on d 14 hepatic genes exhibit rhythmicity during the LDC, consistent with negative-feedback oscillations of *Rev-erb-a* and *Rev-erb-b* mRNA. Muscle appears to coordinate energy availability for transfer in the fetoplacental unit on d 14 of pregnancy, with lower gene expression levels and absence of rhythmicity on d 7 of pregnancy. The switching between d 7 and 14 in the muscle is regulated by *Rev-erb-b*. Muscle activities coincide with a peak of TG/FA levels and lipid transport genes in the fetoplacental unit from ZT12 onward, consistent with a peak expression of placental clock genes toward the end of the light phase or during the dark phase. TG, triglycerides; FA, fatty acids. *B*) Placental lipid homeostasis in human pregnancy. Despite the absence of placental rhythmicity in both early (CVS) and term pregnancies, diurnal fluctuations of triglycerides during the day of normal pregnancy are lost in pregnancies with LGA infants where triglycerides are consistently increased. The melted-clock image denotes uncoupling of metabolic actions from the circadian clock machinery, whereas the normal light and dark phase clocks represent synchronization of metabolic responses with the circadian clocks.

In summary, our data indicate that nutrient accumulation and storage in early pregnancy is achieved by increased metabolic activity of the liver and is accompanied by a “switch on” of metabolic pathways mediated by the muscle and placenta later in pregnancy to regulate energy availability and transfer to the fetus. Our data indicate that anabolic processes in early pregnancy are partially achieved by decoupling from the typical hepatic clock system. They are also consistent with reprogramming of the hepatic and muscle–placenta rhythmic oscillations to coordinate fetal growth in the catabolic phase that characterizes later pregnancy (**Fig. 6*A***). Our human data demonstrate that triglyceride availability and transfer are diurnally programmed in normal pregnancy and disruption of triglyceride oscillations are associated with LGA infants (Fig. 6*B*) and may be related to the pathology of macrosomia.

## Abbreviations

BMI: body mass index
CVS: chorionic villus sampling
FA/TG: fatty acid/triglyceride
FFA: free fatty acid
GDM: gestational diabetes mellitus
LDC: light–dark cycle
LGA: large for gestational age
SCN: suprachiasmatic nucleus
WAT: white adipose tissue
ZT: zeitgeber time
